# Effects of climate change on the distribution of wild *Akebia trifoliata*

**DOI:** 10.1002/ece3.8714

**Published:** 2022-03-23

**Authors:** Jun‐Ming Zhang, Xiang‐Yong Peng, Min‐Li Song, Zhen‐Jian Li, Xin‐Qiao Xu, Wei Wang

**Affiliations:** ^1^ The Institute of Forestry the Chinese Academy of Forestry Beijing China; ^2^ 66340 Department of Biology Taiyuan Normal University Taiyuan China; ^3^ 56650 School of Life Science Qufu Normal University Qufu China; ^4^ 233141 School of Life Sciences Yulin University Yulin China

**Keywords:** ArcGIS, climate change, geographical distribution, MaxEnt, SSR markers, suitable distribution regions

## Abstract

Understanding the impacts and constraints of climate change on the geographical distribution of wild *Akebia trifoliata* is crucial for its sustainable management and economic development as a medicinal material or fruit. In this study, according to the first‐hand information obtained from field investigation, the distribution and response to climate change of *A*. *trifoliata* were studied by the MaxEnt model and ArcGIS. The genetic diversity and population structure of 21 natural populations of *A*. *trifoliata* were studied by simple sequence repeat (SSR) markers. The results showed that the most important bioclimatic variable limiting the distribution of *A*. *trifoliata* was the Mean Temperature of Coldest Quarter (bio11). Under the scenarios SSP1‐2.6 and SSP2‐4.5, the suitable area of *A*. *trifoliata* in the world will remain stable, and the suitable area will increase significantly under the scenarios of SSP3‐7.0 and SSP5‐8.5. Under the current climate scenario, the suitable growth regions of *A*. *trifoliata* in China were 79.9–122.7°E and 21.5–37.5°N. Under the four emission scenarios in the future, the geometric center of the suitable distribution regions of *Akebia trifoliata* in China will move to the north. The clustering results of 21 populations of *A*. *trifoliata* analyzed by SSR markers showed that they had a trend of evolution from south to north.

## Graphical abstract

Precipitation and temperature were the two most important climatic factors that restrict the geographic distribution of *Akebia trifoliata*. Under the current climate scenario, the suitable growth regions of *A*. *trifoliata* in China were 91.7–121.9°E and 21.6–37.5°N. Combined with the evolutionary relationship and prediction results, 21 populations of *A*. *trifoliata* tended to migrate to the north. The distribution center of *A*. *trifoliata* in China will shift to high latitude regions with the increase of temperature in the future.

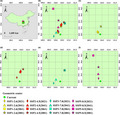

## 1 INTRODUCTION

*Akebia trifoliata* fruit, a popular edible berry in Asia, is widely consumed as daily fruits or functional foods. The fruit of *A*. *trifoliata* is sweet and rich in vitamin C (108–930 mg/100 g), which is beneficial to health and has great value in the third generation of the new fruit industry (Zou et al., 2018, 2019). *A*. *trifoliata* has a long medicinal history in China for more than 2000 years. Its roots, stems, seeds, and fruits, as raw materials of traditional Chinese medicine, have high research value in the field of medicine. In recent years, the demand for *A. trifoliata*, especially as traditional Chinese medicine and fruit, has increased rapidly, resulting in the overexploitation of *A. trifoliata*, the destruction of its wild resources and the loss of its many excellent traits genes. Therefore, the protection and supervision of *A*. *trifoliata* are imminent, which require us to make clear the geographical distribution of *A*. *trifoliata*. Previous studies on *A*. *trifoliata* mainly focused on pharmacological activities (Lu et al., 2019; Wang et al., 2020; Xue et al., 2008) and chemical components (Gao & Wang, 2006; Guan et al., 2008; Iwanaga et al., 2012; Wang et al., 2015), but there is a gap in understanding the natural distribution of *A*. *trifoliata* and its response to climate change.

Due to the progress of human society and global climate change, the ecological environment has been deeply affected. Climate, to a certain extent, determines the spatiotemporal distribution of species, and the change of species’ geographic distribution also reflects the change of climate (Allen & Lendemer, 2016; Descombes et al., 2015). Climate change has a considerable impact on species’ geographic distribution, phenology, and other ecological symptom and progresses, which will lead to the accelerated prosperity or disappearance of species (Acevedo et al., 2020; Lenoir et al., 2008). Understanding how wild species respond to climate change is important for durative protection and supervision of wild species (Yi et al., 2016). Therefore, analyzing the response relationship between climatic variables and species (including *Akebia trifoliata*) is a key step to study the potential geographical distribution change of species, and a necessary way to study the impact of climate change on species survival.

Niche model can be used to predict the potential geographic distribution regions of species and habitat suitability evaluation, which can provide information for ecological research. It is an excellent choice to use the maximum entropy (MaxEnt) model to forecast the potential geographical distribution of species according to the existing species distribution information and various environmental data (Elith & Graham, 2009; Phillips, Anderson, et al., 2006; Phillips & Dudík, 2008. MaxEnt, based on the maximum entropy theory, has good accuracy even when the information on species distribution is insufficient (Saatchi et al., 2008; Yi et al., 2017). The model takes the climatic variables of the existing distribution points of species as the constraint conditions, supposing that the species will appear in all regions with suitable climatic conditions, but not in any regions not suitable for climatic conditions; the greater the entropy of species, the closer the probability distribution of species is to reality (Phillips, Anderson, et al., 2006). At present, MaxEnt has been used to forecast species distribution, and has been applied by many researchers to study the response relationship between species and climatic variables (Abdelaal et al., 2019; Li, Fan, et al., 2020; Yang et al., 2013; Zhang et al., 2019).

In this study, the MaxEnt model was used to predict the suitable distribution of *A*. *trifoliata* under different climate scenarios. The objectives of this study include: (1) to find and evaluate the key climatic variables affecting the distribution of *A*. *trifoliata*; (2) according to the current distribution data, to predict the suitable distribution regions for the growth of *A*. *trifoliata*; (3) to analyze the change trend of suitable distribution regions of *A*. *trifoliata* in different scenarios in the future (Focus on the distribution data concentration region, and the data records mainly occur in China.); (4) according to the prediction results and simple sequence repeat (SSR) markers (The genetic diversity and population structure of 21 *Akebia trifoliata* population were analyzed to determine its evolutionary relationship.), to analyze the migration trend of *A*. *trifoliata* in China.

## 2 MATERIALS AND METHODS

### 2.1 Species occurrence record

**FIGURE 1:**
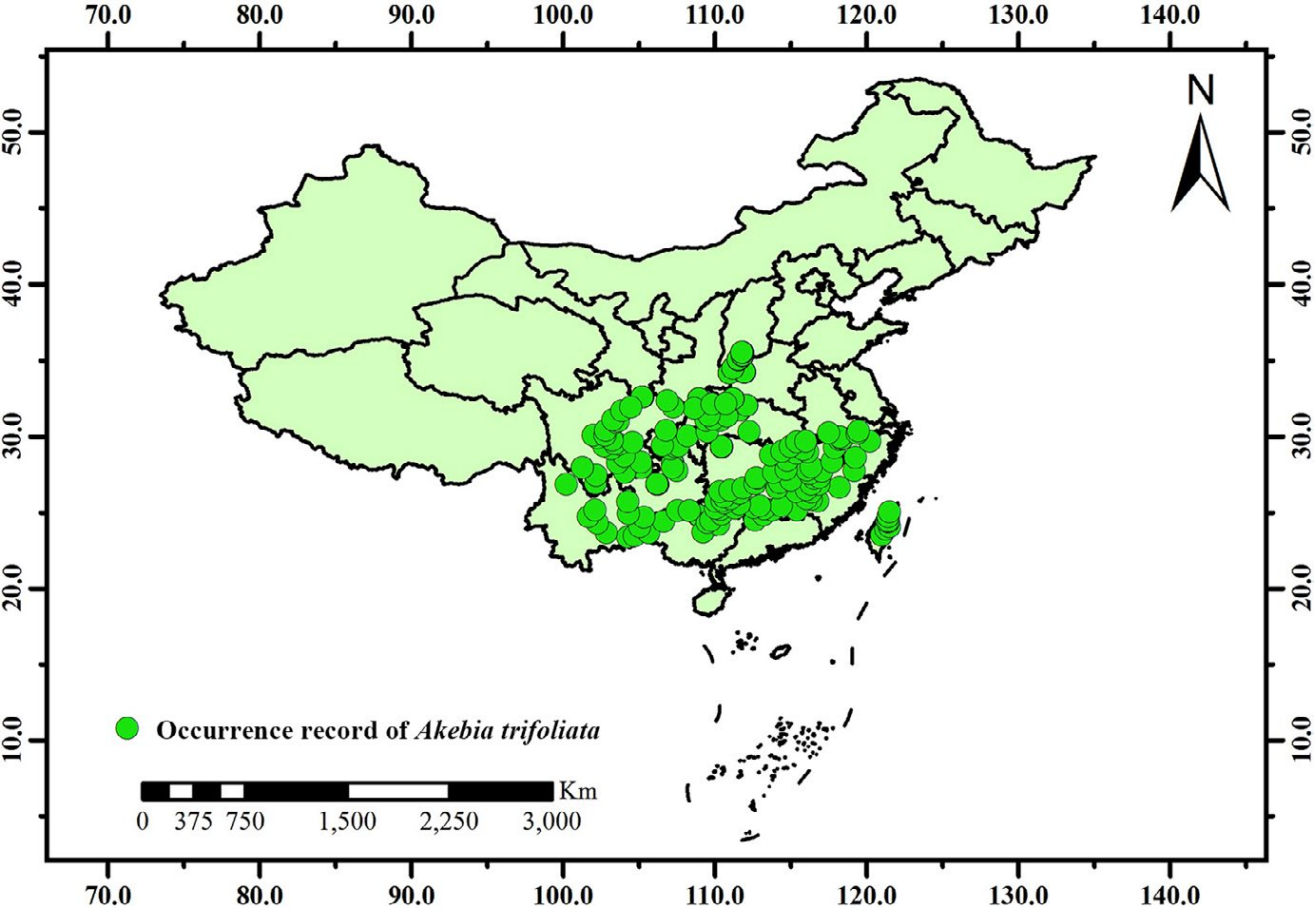
Occurrence record of *Akebia trifoliata* in China

The occurrence record (Figure 1) of *A*. *trifoliata* was obtained from the field survey in 2017–2019 (including 566 points) and downloaded (including 132 points) from the Global Biodiversity Information Facility (GBIF, https://www.gbif.org). The survey covered Northern China, Central China, and Southern China. For the point information from GBIF, we deleted the data lacking longitude and latitude information and fuzzy geographic location information and deleted the wrong or repeated data that cannot be used. Geographical data was gathered from habitats of wild *A*. *trifoliata* by using GPS device (GARMIN GPSMAP 63SC, Kansas City, KS, USA). The actual distribution of *A*. *trifoliata* was analyzed by ArcGIS (version 10.2, ESRI, Redlands, CA, USA) software. The geographic distribution map of richness ×1° was drawn by using a geographic information system (GIS).

### 2.2 Climatic variables

In this study, the climate scenarios used include current climate scenarios (1970–2000), past climate scenarios (the Last Interglacial, the Last Glacial Maximum, and the Mid‐Holocene) (www.worldclim.org/data/v1.4/paleo1.4.html), and future climate scenarios (SSP1‐2.6, SSP2‐4.5, SSP3‐7.0, and SSP5‐8.5) (www.worldclim.org/data/cmip6/cmip6climate.html) (www.carbonbrief.org/cmip6‐the‐next‐generation‐of‐climate‐models‐explained). All climate scenarios contain 19 bioclimatic variables (bio1‐19, the variable value is the average value of the time scale used in the climate scenario) (Table 1), and all climate scenarios were from WorldClim (www.worldclim.org) (Fick & Hijmans, 2017). According to CO_2_ emissions, the four scenarios in the future can be classified as low emission scenario (SSP1‐2.6), medium emission scenario (SSP2‐4.5), medium‐high emission scenario (SSP3‐7.0), and high emission scenario (SSP5‐8.5). The spatial resolution of all climate data is 2.5 minutes.

**TABLE 1:** List of climate variables

Variables	Abbreviation	Units
Annual Mean Temperature	Bio1	℃
Mean Diurnal Range (Mean of monthly (max temp–min temp))	Bio2	℃
Isothermality (BIO2/BIO7) (×100)	Bio3	–
Temperature Seasonality (standard deviation × 100)	Bio4	℃
Max Temperature of Warmest Month	Bio5	℃
Min Temperature of Coldest Month	Bio6	℃
Temperature Annual Range (BIO5‐BIO6)	Bio7	℃
Mean Temperature of Wettest Quarter	Bio8	℃
Mean Temperature of Driest Quarter	Bio9	℃
Mean Temperature of Warmest Quarter	Bio10	℃
Mean Temperature of Coldest Quarter	Bio11	℃
Annual Precipitation	Bio12	mm
Precipitation of Wettest Month	Bio13	mm
Precipitation of Driest Month	Bio14	mm
Precipitation Seasonality (Coefficient of Variation)	Bio15	–
Precipitation of Wettest Quarter	Bio16	mm
Precipitation of Driest Quarter	Bio17	mm
Precipitation of Warmest Quarter	Bio18	mm
Precipitation of Coldest Quarter	Bio19	mm

The time span of each climate scenario in the future is 80 years from 2021 to 2100, which is divided into four time scales: 2021–2040, 2041–2060, 2061–2080, and 2081–2100 (In order to facilitate recording, the time scale of the whole 20 years is marked with the starting year. For example, 2021–2040 is only marked as 2021.). The values of 19 climatic variables are the average values of every 20 years.

### 2.3 Prediction of *Akebia trifoliata* suitable distribution by MaxEnt

In this study, MaxEnt (MaxEnt 3.4.1) was used to predict the potential suitable distribution regions of *A*. *trifoliata* (Phillips, DudíK, et al., 2006). 75% distribution points were randomly selected as training set and 25% as test set. Maximum iterations were set to 5000 to allow the model to have adequate time for convergence. If the model doesn't have enough time to converge (in the form of number of iterations), the model may overpredict or underpredict the relationships. The model was run 10 times to calculate the average value. Other parameters were software default values. The response curve of climatic variables was generated to reflect the relationship between the values of climatic variables and the occurrence probability of species. In addition, the results of the model also include the receiver operator characteristic (ROC) curves, Jackknife test, climatic variable contribution rate statistical table, and so on.

ROC curve analysis is used to verify the accuracy of the MaxEnt model (Wang et al., 2021). In this method, the area under curve, namely AUC value (the value range is 0–1), is used to judge the prediction accuracy of the model. When the AUC value is >0.9, the accuracy of the prediction results is high (Phillips, Anderson, et al., 2006).

In order to avoid the overfitting of the model caused by the collinearity among variables and improve the operation efficiency, the climatic variables were screened after running the full variable prediction (Li, Fan, et al., 2020, 2020; Sillero & Barbosa, 2020). Using the remaining climatic variables after screening, recompile the MaxEnt model, and the calculation efficiency and prediction accuracy of the model will be improved. The screening process was as follows:

1. Calculate the correlation between climatic variables with the Pearson correlation coefficient in SPSS (Statistical Product and Service Solutions, version 26.0, Armonk, NY, USA) software (Table S1).
2. Delete variables (percent contribution is <1%) whose contribution percentage is less than the contribution threshold setting at the first full variable prediction (Table S2). Among the remaining variables with high correlation (the correlation coefficient is >0.8 or <−0.8), the variable with the highest contribution rate is retained as the variable used to recompile the model.

### 2.4 Suitability division of distribution regions of *Akebia trifoliata*

The ASCII file exported by MaxEnt was converted into raster layer by using To Raster tool in ArcGIS, and the raster layer that contains the suitable distribution region of *A*. *trifoliata* was obtained. The fitness value (species existence probability) predicted by the model is continuous raster data, and the range of value is 0–1. The map layer that contains the suitable distribution region was divided into four grades by using the tool of Reclassify of ArcGIS and artificial grading method: no suitability (0 ≤ probability of existence < .15), low suitability (.15 ≤ probability of existence <.33), medium suitability (.33 ≤ probability of existence <.66), and high suitability (probability of existence >.66).

### 2.5 Obtaining the geometric centers of distribution regions

Because the boundary of the suitable distribution regions was irregular, it is difficult to describe the migration of the suitable distribution regions through the change of the boundary. The method of this study was to use the change of geometric centers of suitable distribution regions to describe migration.

Analysis of the geometric centers of the distribution regions of *A*. *trifoliata* involved three tools in ArcGIS: Raster Calculator, Raster Domain, and Mean Center. First, Raster Calculator was used to cut the raster layer that contains the suitable distribution regions of *A*. *trifoliata*, and the raster layer of suitable distribution regions were left. Then, Raster Domain was used to convert the raster layer of suitable distribution regions to plane geometry graphics. Finally, Mean Center was used to find the geometric centers of the plane geometry graphics.

### 2.6 Genetic diversity and population structure of *Akebia trifoliata*

To further reveal the migration status of *A*. *trifoliata* community in China, the genetic diversity and population structure of 21 *A*. *trifoliata* natural populations were studied by using SSR markers (detailed information about SSR primers used in this experiment was shown in Table S3). We used 8 pairs of primers to detect 194 alleles in 578 individuals of 21 natural populations. The distance between individuals should be at least 50 meters to avoid collecting asexually reproduced individuals.

## 3 RESULTS

### 3.1 Performance analysis of the model

**FIGURE 2:**
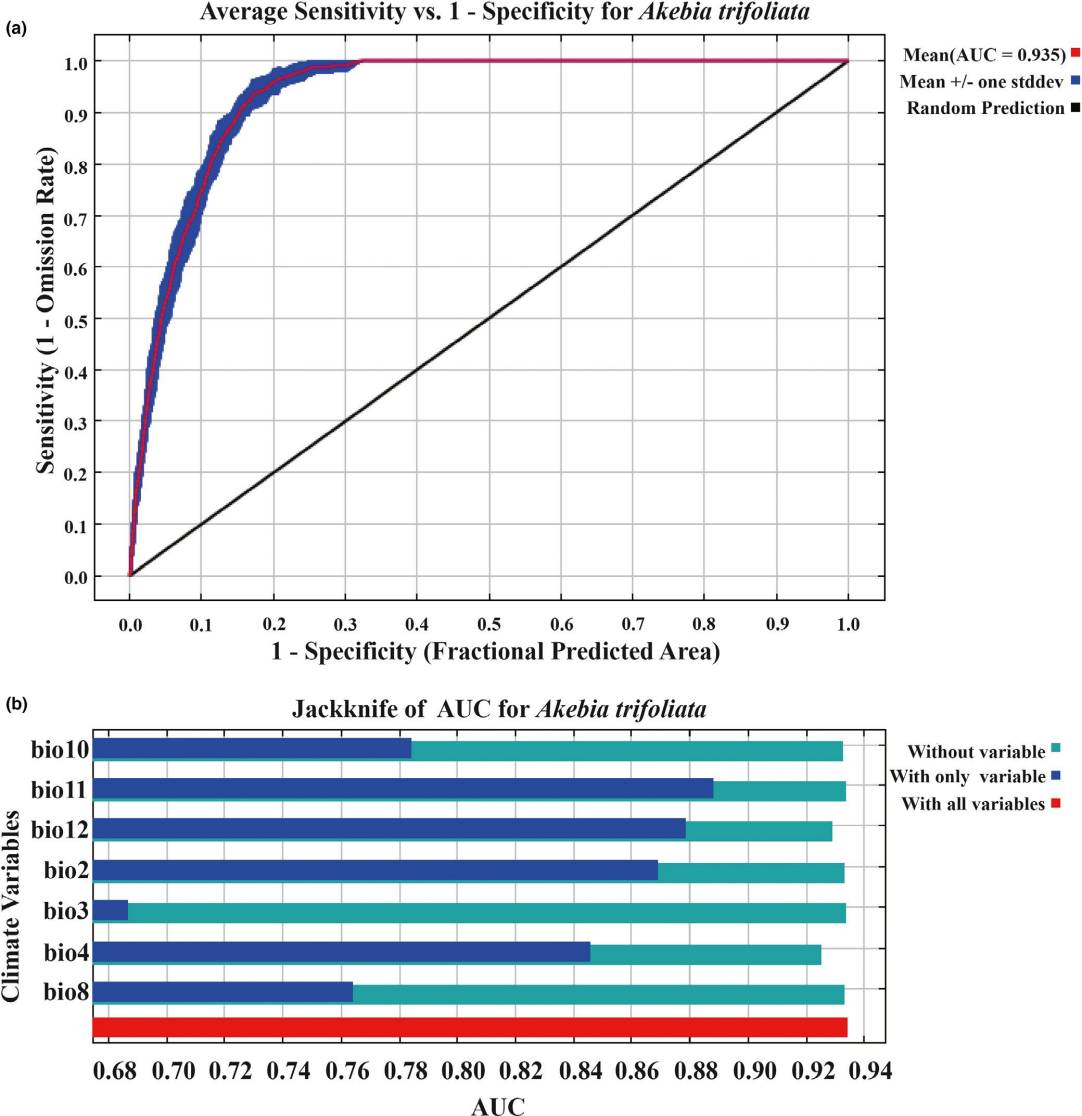
ROC curve, Jackknife test of MaxEnt. (a) ROC curve of the Maxent model (10 runs). (b) Jackknife test of the Maxent model

The average AUC of the prediction results of this model was 0.935 (Figure 2a), which indicates that accuracy of prediction results was high.

### 3.2 Climatic variables analysis

According to the screening mechanism of the method part in this paper, combined with the contribution rate of 19 climatic variables (bio1‐19) in MaxEnt model and the correlation test of them, the remaining 7 climatic variables (bio2, bio3, bio4, bio8, bio10, bio11 and bio12) were used to run MaxEnt model again.

**FIGURE 3:**
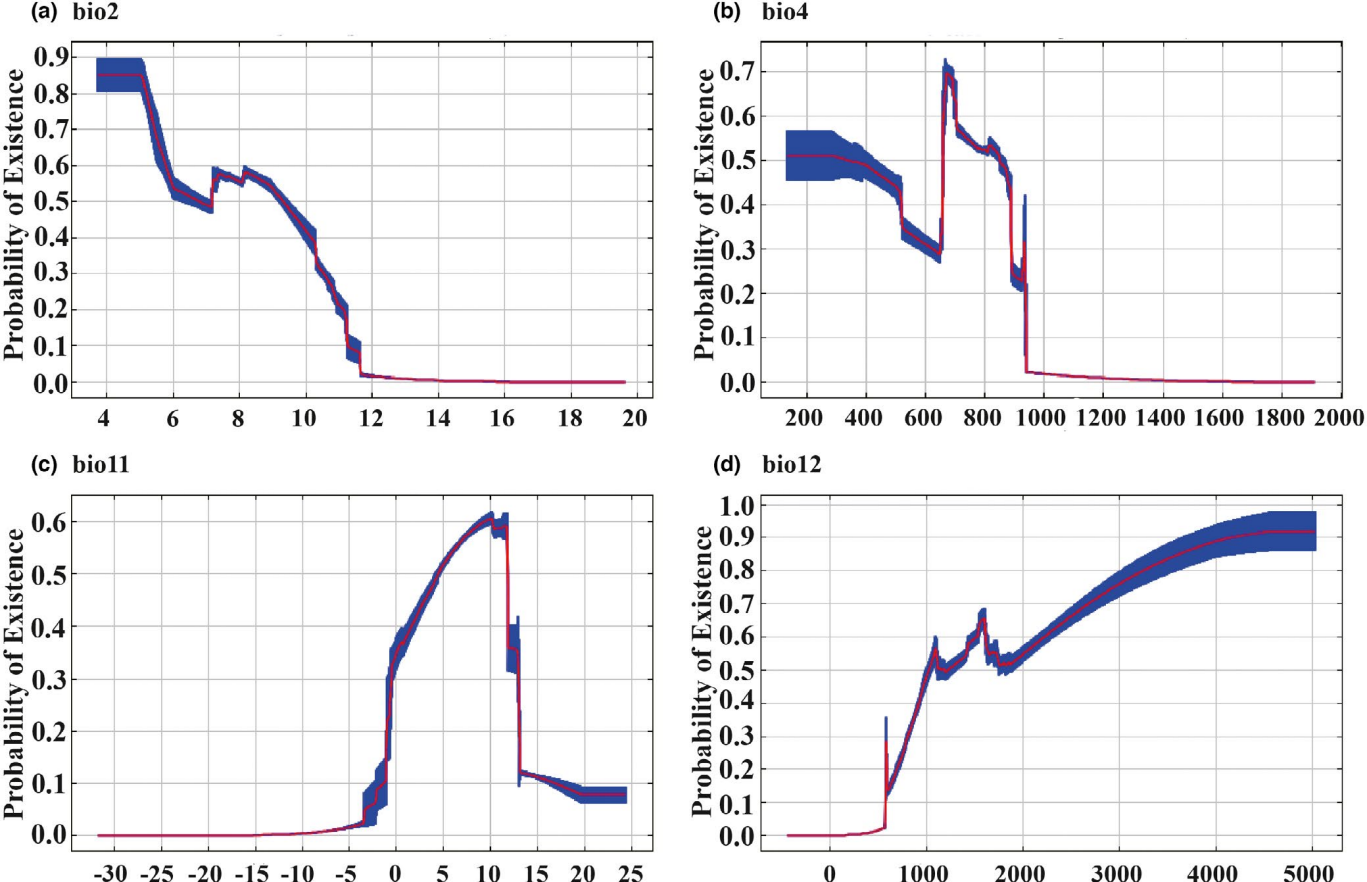
Response curves between the probability of presence and climate variables (10 runs). Blue: mean ±one standard deviation. (a) the Mean Diurnal Range (bio2). (b) the Temperature Seasonality (bio4). (c) the Mean Temperature of Coldest Quarter (bio11). (d) the Annual Precipitation (bio12)

Jackknife test (using AUC on test data) was used to analyze the impact of climatic variables on the prediction results to determine the importance of each climatic factor (Figure 2b). When a climatic variable was used alone, the four variables with greater gain were bio2, bio4, bio11, and bio12. Bio11 has the highest gain value. This shows that the above four climatic variables were the main influencing factors for predicting the suitable distribution regions of *A*. *trifoliata*. The response curves of four important climatic variables and the existence probability of *A*. *trifoliata* were shown in Figure 3.

Bio2, bio4, and bio11 are temperature‐related climatic variables. When bio 2 is less than 14℃, the existence probability of *A*. *trifoliata* is >0, and with the continuous decrease of bio 2, the existence probability shows an overall upward trend, which indicates that stable temperature conditions are more conducive to the survival of *A*. *trifoliata*. When bio4 is equal to 655℃, the existence probability of *A*. *trifoliata* can reach a peak. If bio4 continues to decrease, the existence probability decreases rapidly and then increases slowly. When bio4 is less than 286℃, the existence probability will remain above .5. However, when bio4 increases on the basis of 655℃, the probability of existence will eventually become 0. When bio11 is <−10℃, the existence probability of *A*. *trifoliata* is close to 0. When bio11 is >−0℃, the existence probability begins to increase and reaches the peak at 10℃. From this point, the existence probability decreases rapidly with the continuous increase of bio11. Bio12 is a precipitation related variable. When bio12 is <400 mm, the existence probability of *A*. *trifoliata* is close to 0. Then, with the continuous increase of bio12, the existence probability also increases. When bio12 reaches 4500 mm, the existence probability can reach more than 0.9.

### 3.3 Geographical distribution prediction of *Akebia trifoliata*

#### 3.3.1 Current suitable distribution regions

**FIGURE 4:**
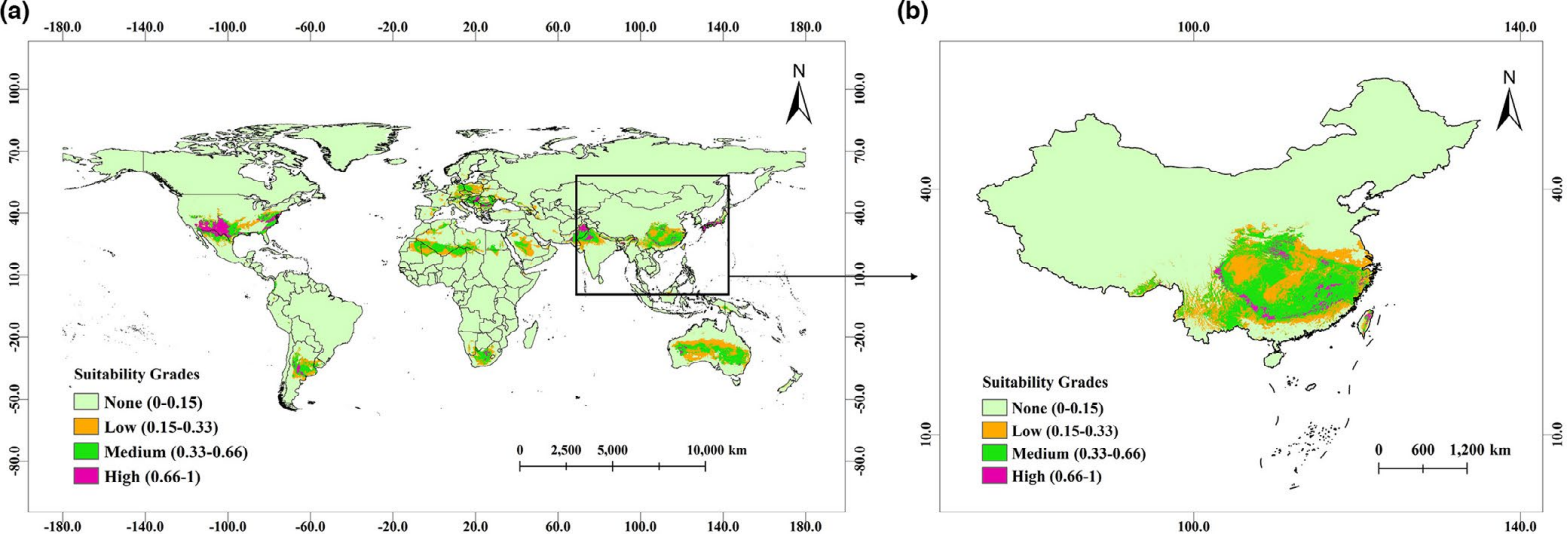
Current suitable distribution regions of *Akebia trifoliata*. (a) Distribution of *Akebia trifoliata* in the world. (b) Distribution of *Akebia trifoliata* in China

Figure 4a shows the suitable distribution regions of *A*. *trifoliata* in the world, and Figure 4b shows the suitable distribution regions of *A*. *trifoliata* in China. All the prediction results were predicted according to the distribution data of *A*. *trifoliata* in China.

In the world model, the total suitable area of *A*. *trifoliata* is 1690.88 × 10^4^ km^2^, and the suitable distribution regions is mainly distributed in the temperate zone and less in tropical zone. In China, the suitable growth regions of *A*. *trifoliata* are 79.9–122.7°E and 21.6–37.6°N (the suitable distribution regions are basically consistent with Figure 1), and the total suitable area is 192.01 × 10^4^ km^2^. The detailed values of suitable area in the world were shown in Tables S4, and S5 shows the detailed values of suitable area in China.

#### 3.3.2 Suitable distribution regions of *Akebia trifoliata* in the past

Figure S1 shows the suitable distribution regions of *A*. *trifoliata* in the past. In the Last Interglacial scenario (Figure S1a), the total suitable area of *A*. *trifoliata* was 661.15 × 10^4^ km^2^. In the Last Glacial Maximum scenario (Figure S1b), the total suitable area was 910.81 × 10^4^ km^2^. The total suitable area in these two scenarios was much smaller than that in the current scenario. In the Mid‐Holocene scenario (Figure S1c), the total suitable area was 1596.03 × 10^4^ km^2^, slightly different from the total suitable area under the current scenario. However, in the Mid‐Holocene scenario, the suitable distribution regions were mainly concentrated in the tropics. The detailed values of suitable area in the past were shown in Table S6.

#### 3.3.3 Suitable distribution regions of *Akebia trifoliata* in the future

**FIGURE 5:**
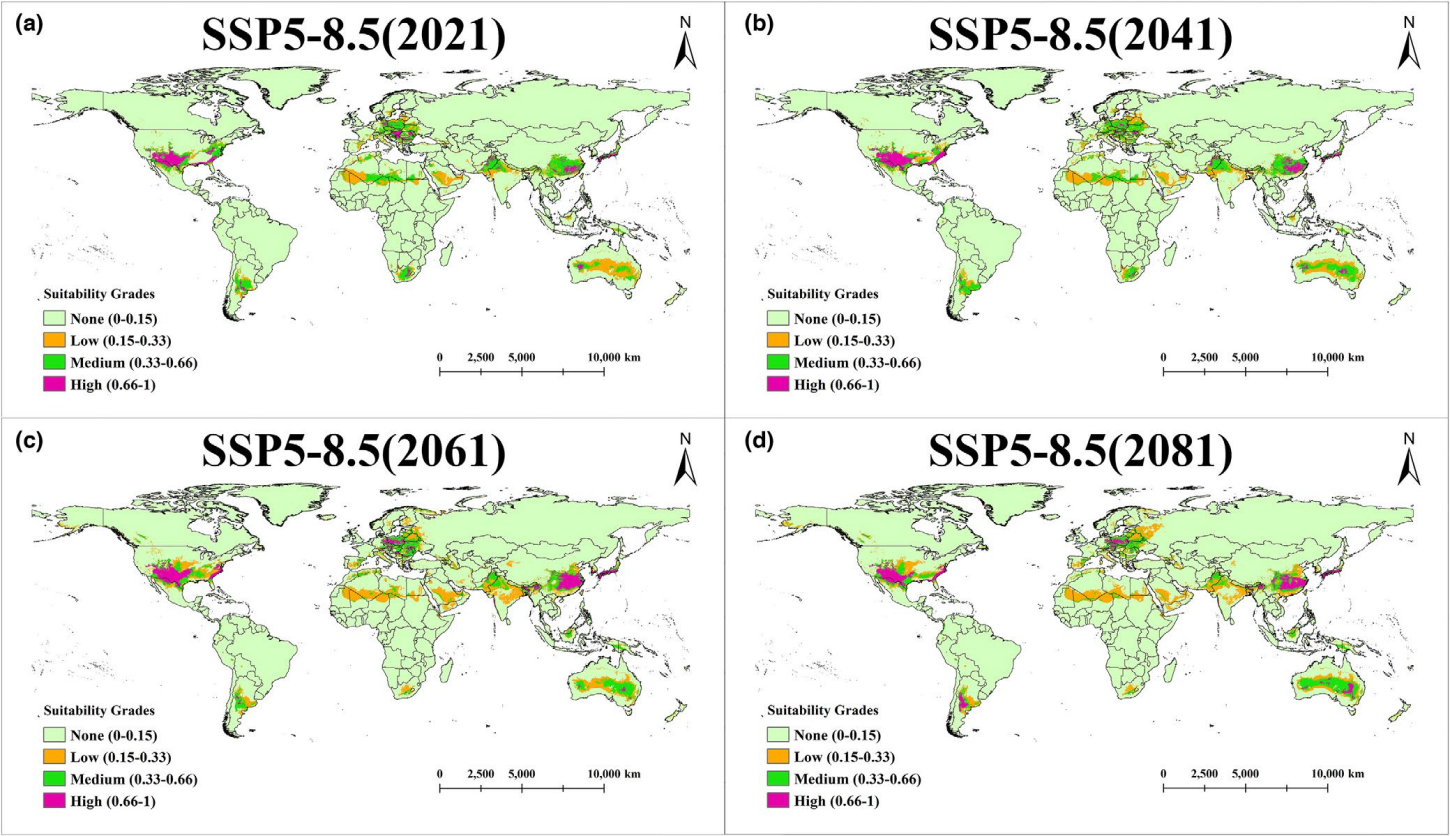
The suitable distribution regions of *Akebia trifoliata* in scenario SSP5‐8.5. (a) The suitable distribution regions in 2021. (b) The suitable distribution regions in 2041. (c) The suitable distribution regions in 2061. (d) The suitable distribution regions in 2081

The prediction of suitable distribution regions of *A*. *trifoliata* under four emission scenarios (SSP1‐2.6, SSP2‐4.5, SSP3‐7.0 and SSP5‐8.5) was shown in Figures 5 and [Link], [Link], [Link]. The detailed values of suitable area in the world were shown in Tables S4 and S5 shows the detailed values of suitable area in China.

**FIGURE 6:**
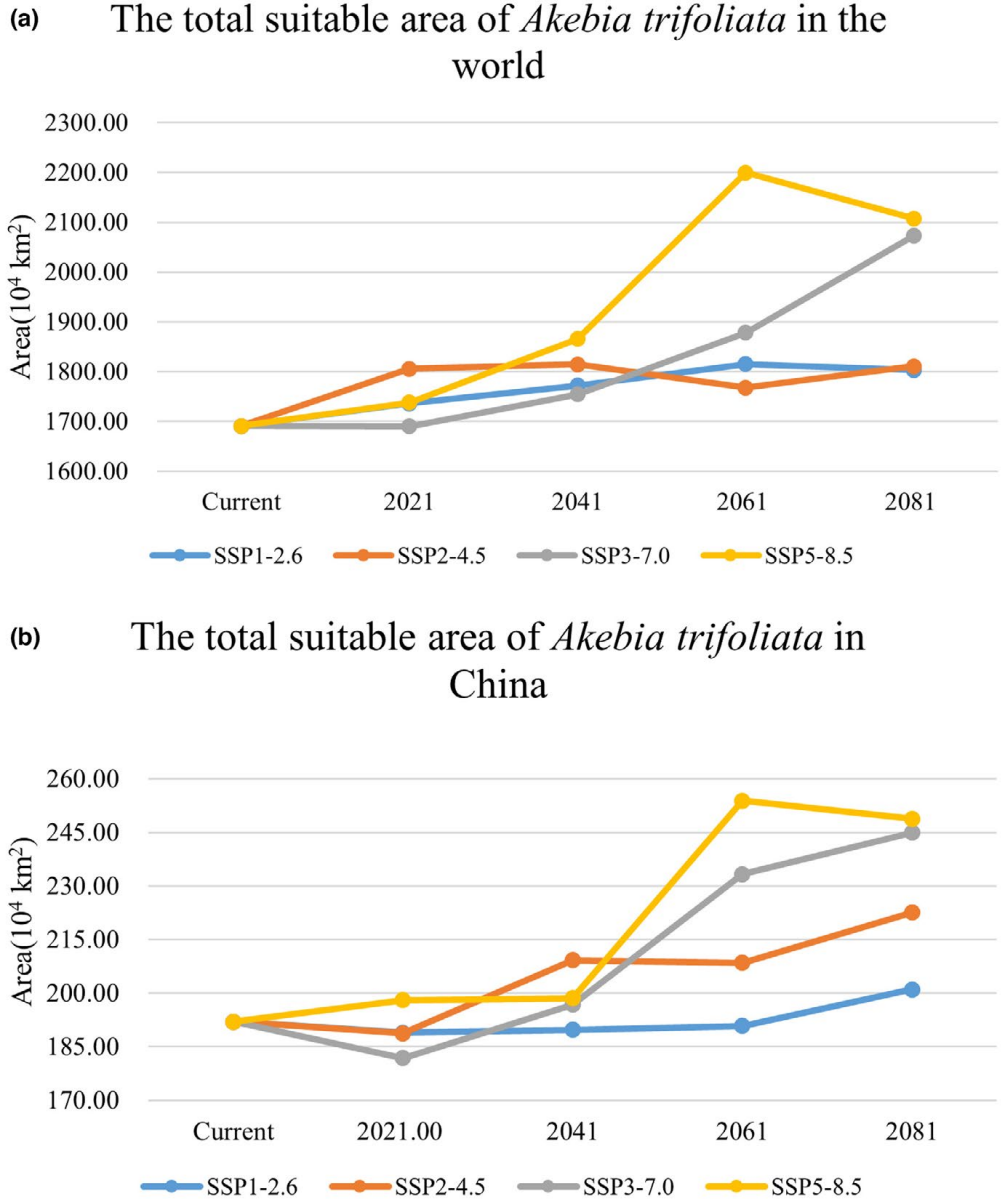
The broken line diagram of the suitable area of *Akebia trifoliata*. (a) The total suitable area of *Akebia trifoliata* in the world. (b) The total suitable area of *Akebia trifoliata* in China

According to the broken line diagram of the suitable area of *A*. *trifoliata* in the world (Figure 6a), it is found that the suitable area of this species fluctuates slightly in emission scenarios SSP1‐2.6 and SSP2‐4.5. In scenario SSP3‐7.0, the suitable area increases significantly after 2041. In scenario SSP5‐8.5, the change of suitable area is the most obvious, and the suitable area increases greatly in 2041 and 2061 and begins to decrease after 2061.

In China, the suitable area of *A*. *trifoliata* remains stable in scenario SSP1‐2.6 (Figure 6b), and only increases slightly in the interval between 2061 and 2081. In the other three scenarios, the change of suitable area is larger. Overall, the range of change of suitable area increases with the upgrading of emission scenarios.

### 3.4 Geometric centers of suitable distribution regions of *Akebia trifoliata*

**FIGURE 7:**
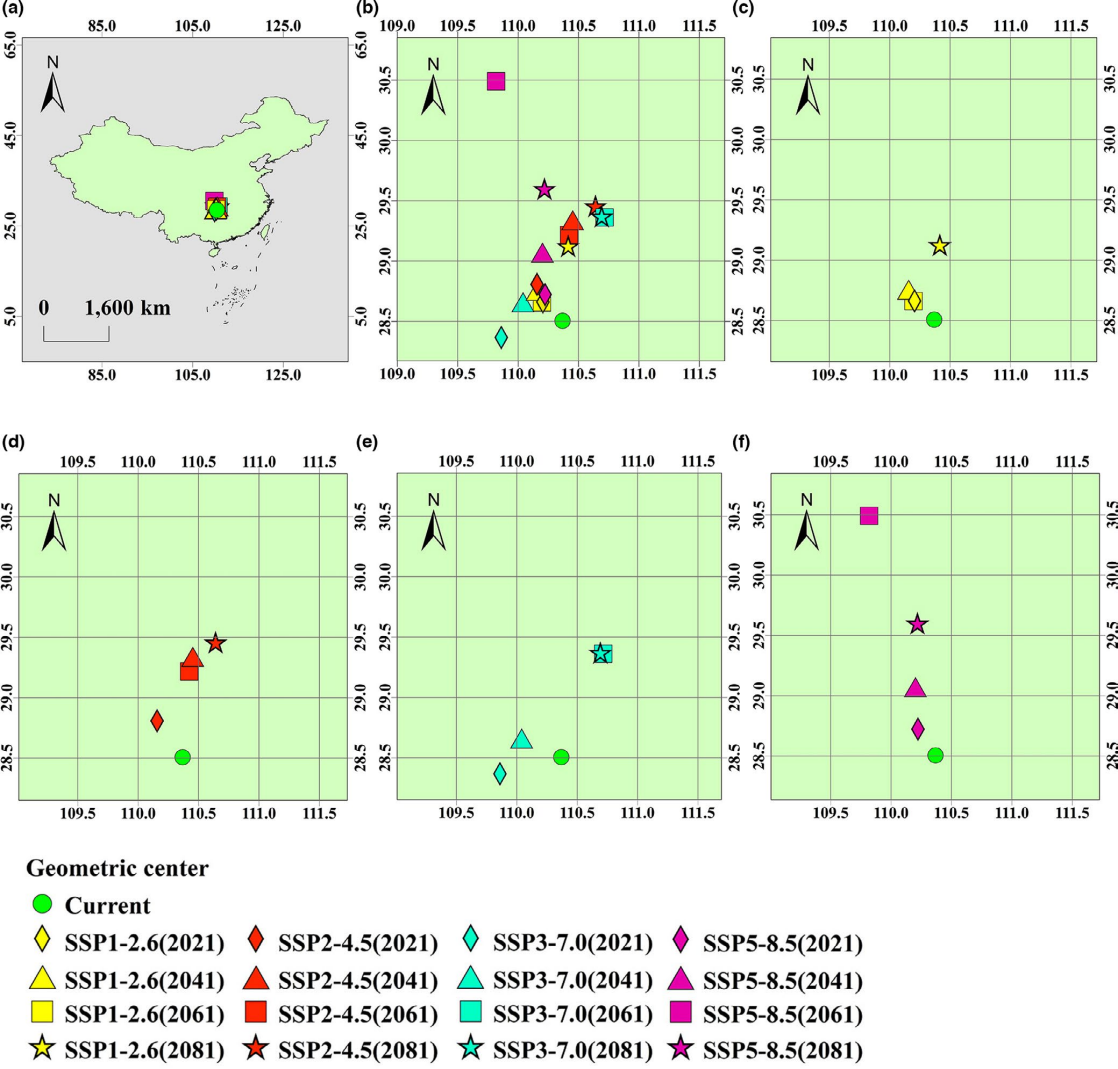
Geometric centers of suitable distribution regions of *Akebia trifoliata* in China. (a–b) Geometric centers under current scenario and four future scenarios (enlarge a to get b). (c–f) Geometric centers in SSP1‐2.6, SSP2‐4.5, SSP3‐7.0 and SSP5‐8.5 scenarios

Figure 7 shows the distribution of geometric centers of suitable distribution regions of *Akebia trifoliata* in China under five scenarios (including geometric centers under the current scenario and four future scenarios). Enlarge Figure 7a to get 7b. In scenario SSP1‐2.6 (Figure 7c), the geometric centers in 2021, 2041, and 2061 were in the northwest of the original geometric center (current scenario), and the migration distance is small. In 2081, the geometric center moved to the northeast of the original geometric center, with a large migration distance. The fluctuation range is 28.51–29.13°N, and the fluctuation value is 0.62° (fluctuation upper limit minus lower limit).

In scenario SSP2‐4.5, the fluctuation range of geometric centers were 28.51–29.49°N (Figure 7d), and the fluctuation value was 0.98°. In scenario SSP3‐7.0, the fluctuation range of the geometric centers were 28.37–29.37°N (Figure 7e), and the fluctuation value was 1.00°. In scenario SSP5‐8.5, the fluctuation range of the geometric centers were 28.51–29.60°N (Figure 7f), and the fluctuation value was 1.09°.

With the upgrading of emission scenarios, the fluctuation value of geometric center is also increasing, which indicates that the change of suitable distribution regions of *A*. *trifoliata* in high emission scenario is more active than that in low emission scenario. In addition, in scenario SSP3‐7.0 in 2021, the geometric center moves to the south of the original geometric center, but all the other geometric centers are in the north of the original geometric center. Overall, the suitable distribution regions of *A*. *trifoliata* in China may migrate to the north in the future.

### 3.5 Clustering results of 21 natural populations of *Akebia trifoliata*

**FIGURE 8:**
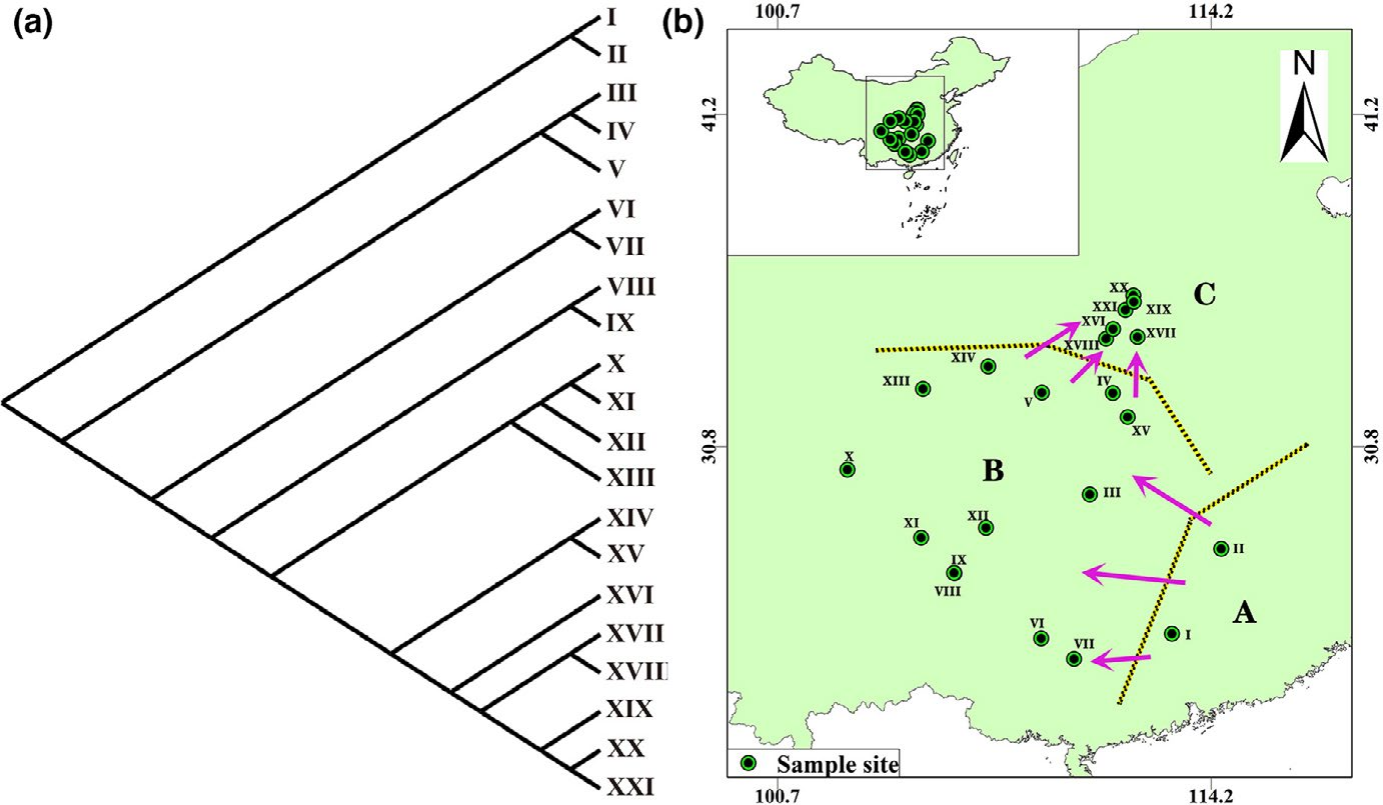
Clustering results and evolutionary relationship of 21 *Akebia trifoliata* populations. (a) Clustering results by Neighbor‐joining Method of 21 *Akebia trifoliata* populations. (b) Distribution of 21 *Akebia trifoliata* populations

The clustering results of 21 *A*. *trifoliata* populations by neighbor‐joining method revealed the evolutionary relationship of these 21 populations, as shown in Figure 8a. The geographic location information of these 21 populations was shown in Figure 8b. These 21 populations can be divided into three parts: A (I and II), B (III–XV), and C (XVI–XXI). In these three parts, the genetic relationship of A is closer to the ancestor, B is farther from the ancestor, and C is farthest from the ancestor. The genetic relationship among these three parts and ancestor may be the result of ancestral population migration. The migration direction was shown by the purple arrow in Figure 8b (from Southeast China to Southwest China and Central China, and then to Northern China).

## 4 DISCUSSION

### 4.1 Main climatic variables affecting the distribution of *Akebia trifoliata*

Temperature and precipitation are two key factors limiting plant growth. When the climate factors related to temperature and precipitation change too much and exceed the current threshold of plant growth, it will lead to the migration of its population (Camille & Gary, 2003). Water is particularly important for plants. It not only has a significant impact on the photosynthesis of plants, but also determines the growth status of plants. Therefore, precipitation is an important factor limiting the distribution of plants (Zhang et al., 2018). The longer growing season is helpful for the plant population to migrate to more suitable regions, and the increase of precipitation in the driest month can prolong the growth season (Vaganov et al., 1999). Extreme low and high temperatures also limit the growth of plants. Plants will suffer from freezing damage if they are exposed to low temperature for a long time, and the decrease of the lowest temperature in the coldest month will undoubtedly aggravate the damage (Harsch & HilleRisLambers, 2016). However, high temperature will destroy the water balance in plants, leading to protein condensation and accumulation of harmful metabolites (Lemmens et al., 2006). If the highest temperature in the warmest month increases, the growth of plants will be hindered to a certain extent.

In this study, the 19 variables used to predict the distribution of species belong to the category of temperature and precipitation factors. The results of Jackknife test showed that bio2, bio4, bio11, and bio12 were the four key variables limiting the distribution of *A*. *trifoliata*. Among them, the most critical variable is bio11, which belongs to the variable related to temperature factor. The research on *A*. *quinata* shows that the key variable limiting its distribution is bio12 (Zhang et al., 2021), which is related to precipitation factors. This indicates that the priority of temperature conditions on the restriction of *A*. *trifoliata* may be higher than that of precipitation conditions, and the priority of precipitation condition to the limitation of *A*. *quinata* may be higher than that of temperature condition.

### 4.2 Change trend of suitable distribution regions of *Akebia trifoliata*

Species respond differently to climate change. In the future, the suitable area of *A*. *trifoliata* in the world will tend to be stable under low emission scenarios (SSP1‐2.6) and medium emission scenarios (SSP2‐4.5). In the medium‐high emission scenarios (SSP3‐7.0) and high emission scenarios (SSP5‐8.5), the suitable area has increased significantly. In the study of two species of peony (Zhang et al., 2018), the suitable area of *Paeonia rockii* was increased in both of emission scenario (RCP2.6) and emission scenario (RCP8.5) (for a detailed description of the climate scenario, see www.carbonbrief.org/cmip6‐the‐next‐generation‐of‐climate‐models‐explained). The research on *Tricholoma matsutake* shows that in the scenario RCP8.5, its suitable area will be greatly reduced, and the distribution regions will even be fragmented (Guo et al., 2017). Under the four different emission scenarios in the future, the geometric center of the suitable distribution regions of *A*. *quinata* in East Asia continues to move to the northeast with the upgrading of the emission scenario, which is caused by the continuous reduction of the suitable area in China (Zhang et al., 2021). Due to climate change, the suitable distribution area of species will also change (Hampe & Petit, 2005; Ramírez‐Preciado et al., 2019; Thuiller et al., 2008). The expansion or contraction of species distribution region, species migration, and even the fragmentation of distribution region is the reason for the change of geometric center of species distribution area (Li, Li, et al., 2020, 2020; Zhang et al., 2021). In the future scenario, the geometric centers of the suitable distribution regions of *A*. *trifoliata* move north, which may be because it has the trend of northward expansion.

### 4.3 Analysis on evolutionary relationship of *Akebia trifoliata* population

The genetic diversity of plant populations is closely related to the climatic factors of their habitats (Eckert et al., 2010; Howe et al., 2003; Nybom, 2004; Turpeinen et al., 2001; Wróblewska, 2014). The process of species migration and adaptation to new habitats must be accompanied by changes in climate factors, which will promote the genetic variation of species. The evolutionary relationship of 21 *A*. *trifoliata* populations in China may be related to the change of habitat during the migration of this species.

## 5 CONCLUSIONS

Predicting how climate change will affect the distribution of *A*. *trifoliata* is of great significance for its conservation. The Mean Diurnal Range (bio2), the Temperature Seasonality (bio4), the Mean Temperature of Coldest Quarter (bio11), and the Annual Precipitation (bio12) are the main climatic variables that affect the distribution of *A*. *trifoliata*. In the future, the suitable area of *A*. *trifoliata* in the world will remain stable in low emission scenario (SSP1‐2.6) and medium emission scenario (SSP2‐4.5) and increase significantly in medium‐high and high emission scenarios (SSP3‐7.0 and SSP5‐8.5). The geometric center of the distribution area of *A*. *trifoliata* in China will move to the north. There was a migration trend of *A*. *trifoliata* in China from south to north.

## CONFLICT OF INTEREST

The authors report no conflict of interest.

## AUTHOR CONTRIBUTIONS

**Jun‐Ming Zhang:** Writing – original draft (equal). **Xiang‐Yong Peng:** Data curation (equal); Investigation (equal). **Min‐Li Song:** Writing – review & editing (equal). **Zhen‐Jian Li:** Investigation (equal); Writing – review & editing (equal). **Xin‐Qiao Xu:** Project administration (equal); Writing – review & editing (equal). **Wei Wang:** Data curation (equal); Investigation (equal); Project administration (equal); Writing – original draft (equal).

## Supporting information

Fig S1

Fig S2

Fig S3

Fig S4

Supplementary Material

Table S1

Table S2

Table S3

Table S4

Table S5

Table S6

## Data Availability

The original contributions presented in the study are included in the article/[Link], [Link], [Link], [Link], [Link], [Link], [Link], [Link], [Link], [Link], [Link]; further inquiries can be directed to the corresponding author/s.
