# Supplementary figures and images for: Effects of climate change on the distribution of wild Akebia trifoliata
Source: Ecol Evol. 2022 Mar 23;12(3):e8714. doi: 10.1002/ece3.8714 (PMC8941373; doi:10.1002/ece3.8714)

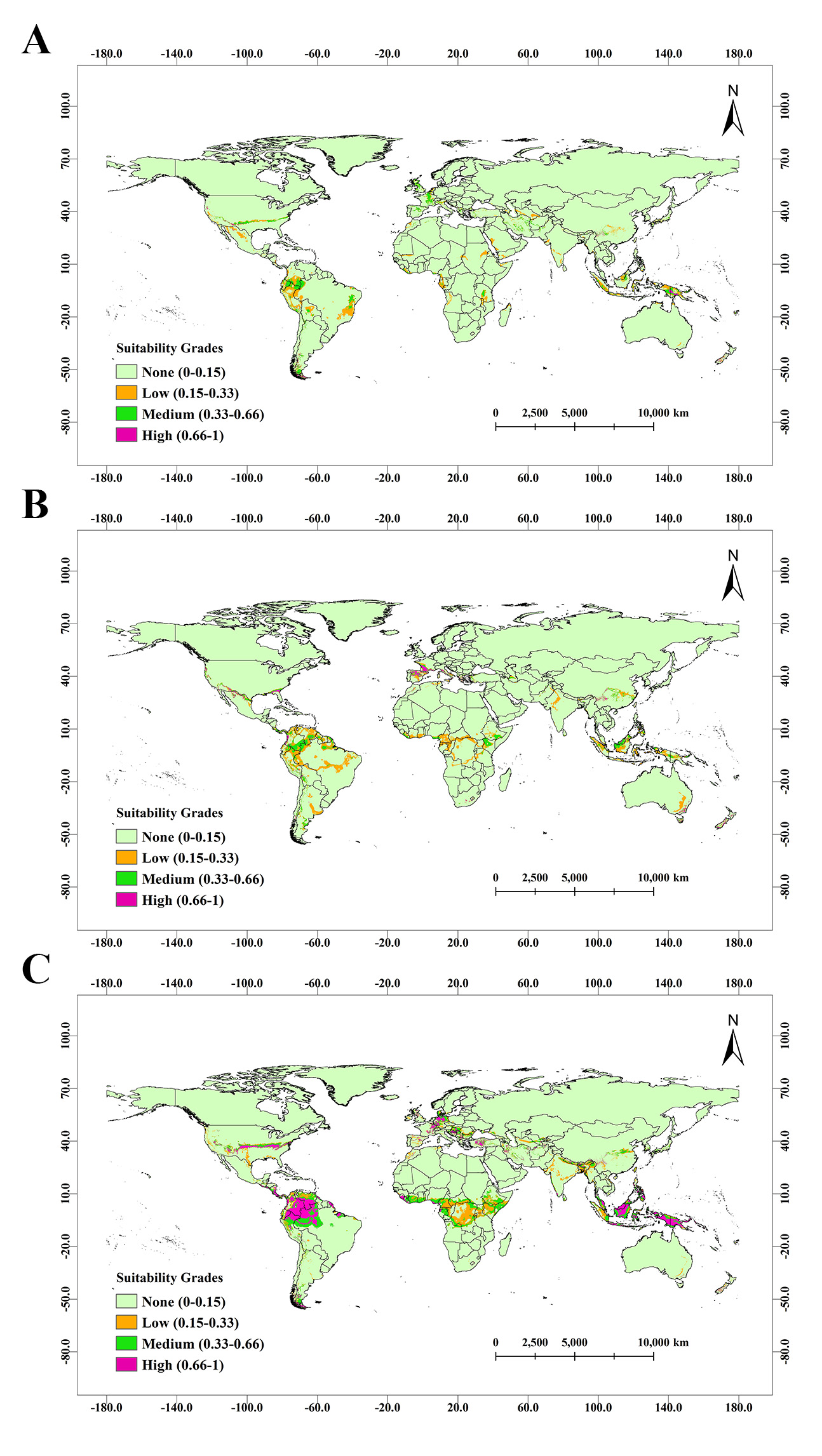

Supplement: Supplementary file 1 — Fig S1 [file ECE3-12-e8714-s010.jpg]

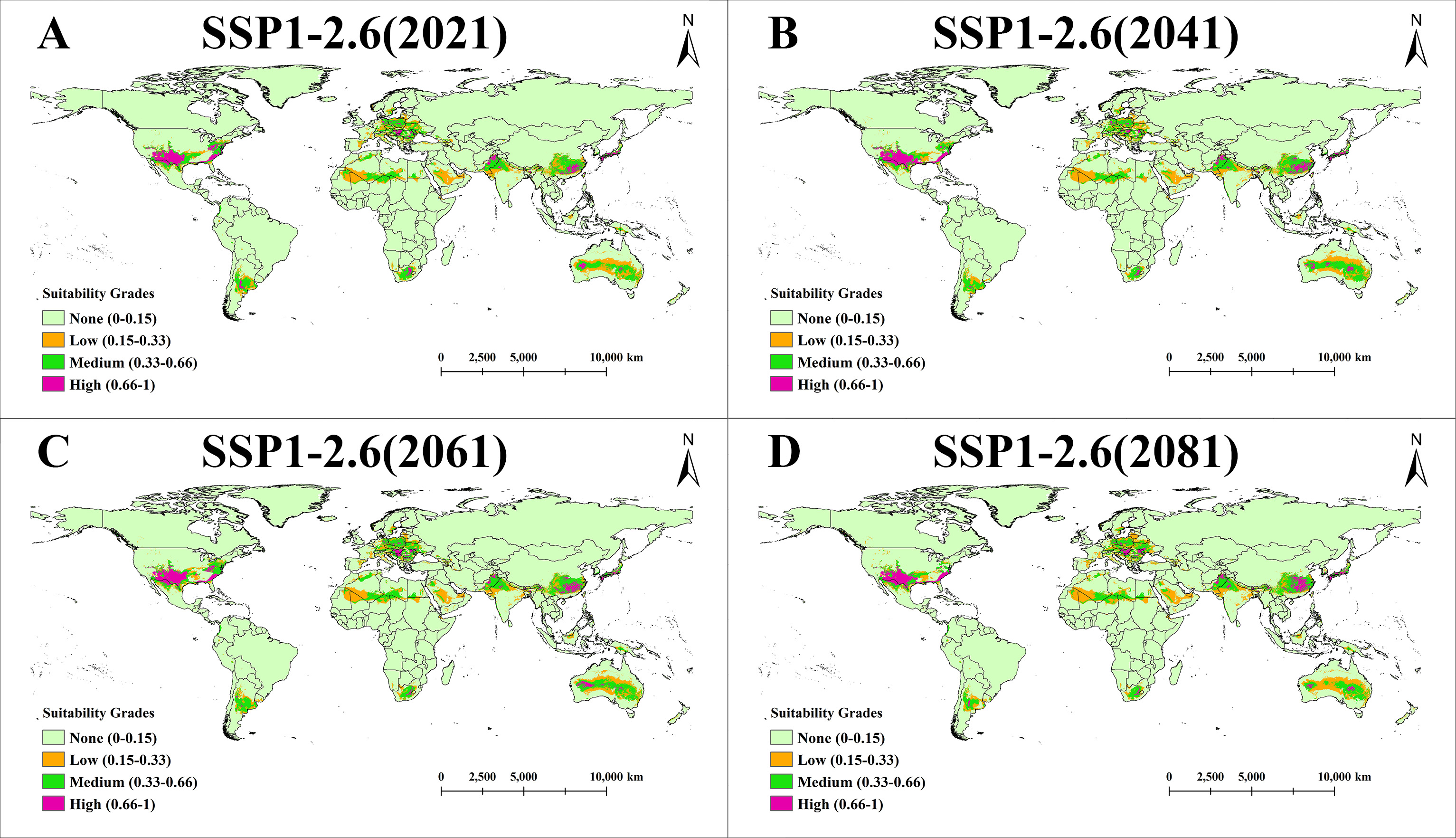

Supplement: Supplementary file 2 — Fig S2 [file ECE3-12-e8714-s007.jpg]

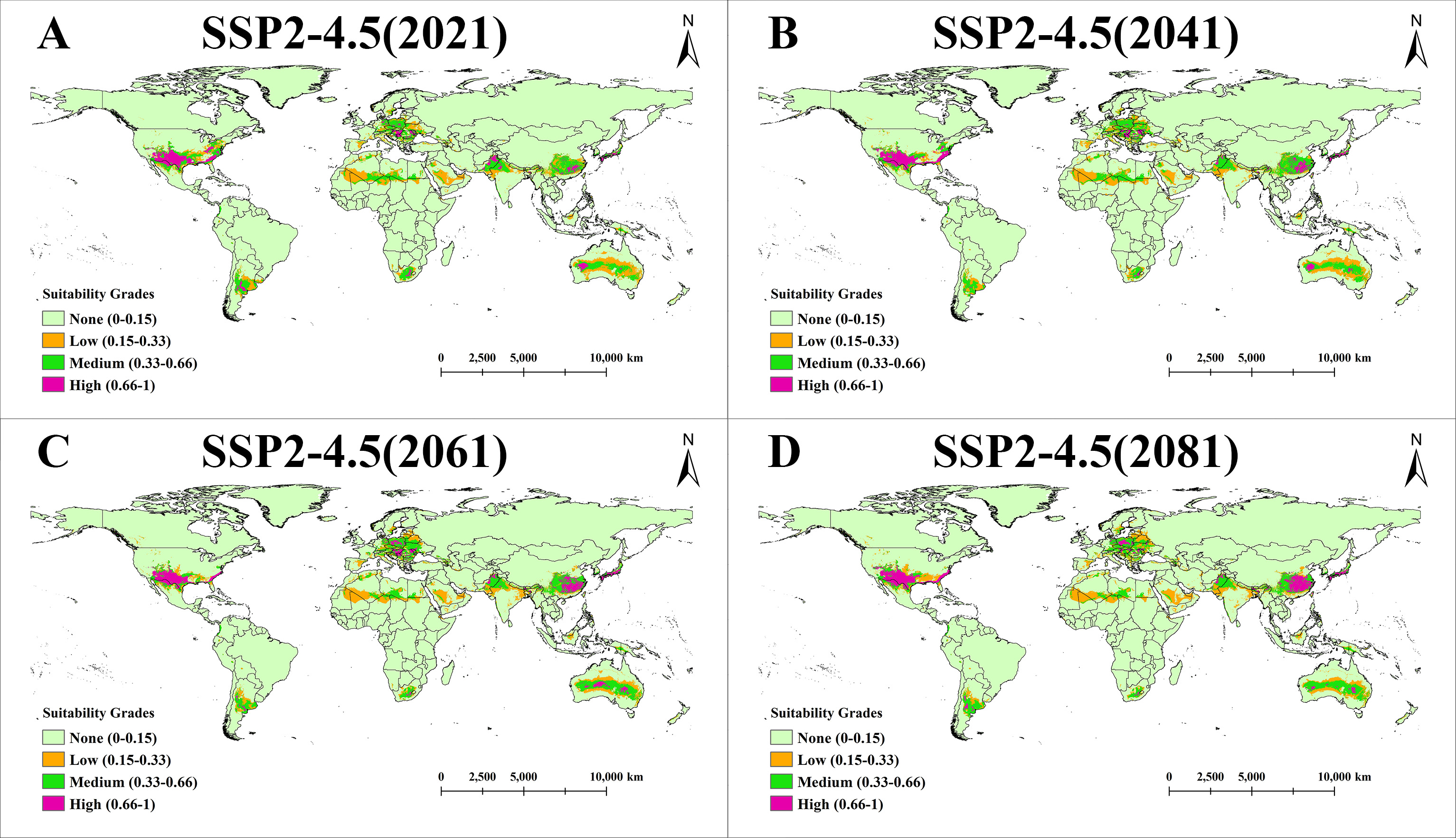

Supplement: Supplementary file 3 — Fig S3 [file ECE3-12-e8714-s008.jpg]

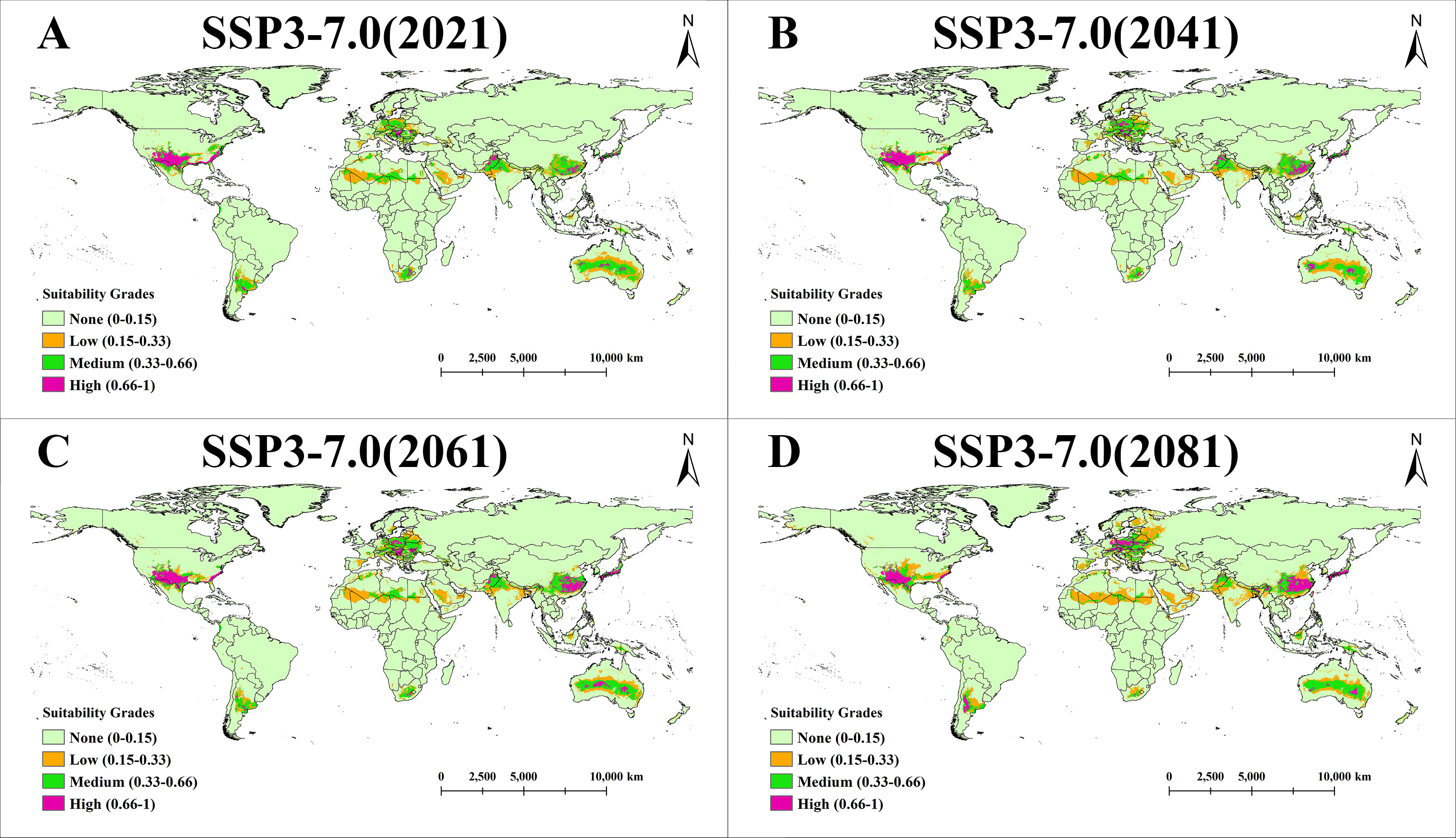

Supplement: Supplementary file 4 — Fig S4 [file ECE3-12-e8714-s003.jpg]
