# Supplementary material for: Effects of climate change on the distribution of wild Akebia trifoliata
Source: Ecol Evol. 2022 Mar 23;12(3):e8714. doi: 10.1002/ece3.8714 (PMC8941373; doi:10.1002/ece3.8714)
Supplement: Supplementary file 5 — Supplementary Material [file ECE3-12-e8714-s004.doc]

Figure S1. The suitable distribution regions of *Akebia trifoliata* in the past. A: The suitable distribution regions in the Last Interglacial. B: The suitable distribution regions in the Last Glacial Maximum. C: The suitable distribution regions in the Mid Holocene.

Figure S2. The suitable distribution regions of *Akebia trifoliata* in scenario SSP1-2.6. A: The suitable distribution regions in 2021. B: The suitable distribution regions in 2041. C: The suitable distribution regions in 2061. D: The suitable distribution regions in 2081.

Figure S3. The suitable distribution regions of *Akebia trifoliata* in scenario SSP2-4.5. A: The suitable distribution regions in 2021. B: The suitable distribution regions in 2041. C: The suitable distribution regions in 2061. D: The suitable distribution regions in 2081.

Figure S4. The suitable distribution regions of *Akebia trifoliata* in scenario SSP3-7.0. A: The suitable distribution regions in 2021. B: The suitable distribution regions in 2041. C: The suitable distribution regions in 2061. D: The suitable distribution regions in 2081.
