# Supplementary material for: Effects of climate change on the distribution of wild Akebia trifoliata
Source: Ecol Evol. 2022 Mar 23;12(3):e8714. doi: 10.1002/ece3.8714 (PMC8941373; doi:10.1002/ece3.8714)
Supplement: Supplementary file 6 — Table S1 [file ECE3-12-e8714-s006.docx]

Table S1 The correlation coefficients between the climate variables

| Variable | BIO1 | BIO2 | BIO3 | BIO4 | BIO5 | BIO6 | BIO7 | BIO8 | BIO9 | BIO10 | BIO11 | BIO12 | BIO13 | BIO14 | BIO15 | BIO16 | BIO17 | BIO18 | BIO19 |
| --- | --- | --- | --- | --- | --- | --- | --- | --- | --- | --- | --- | --- | --- | --- | --- | --- | --- | --- | --- |
| BIO1 | 1 |  |  |  |  |  |  |  |  |  |  |  |  |  |  |  |  |  |  |
| BIO2 | -0.580 | 1 |  |  |  |  |  |  |  |  |  |  |  |  |  |  |  |  |  |
| BIO3 | -0.198 | 0.498 | 1 |  |  |  |  |  |  |  |  |  |  |  |  |  |  |  |  |
| BIO4 | -0.417 | 0.606 | -0.378 | 1 |  |  |  |  |  |  |  |  |  |  |  |  |  |  |  |
| BIO5 | 0.700 | -0.119 | -0.497 | 0.349 | 1 |  |  |  |  |  |  |  |  |  |  |  |  |  |  |
| BIO6 | 0.896 | -0.818 | -0.143 | -0.731 | 0.353 | 1 |  |  |  |  |  |  |  |  |  |  |  |  |  |
| BIO7 | -0.513 | 0.780 | -0.149 | 0.969 | 0.232 | -0.829 | 1 |  |  |  |  |  |  |  |  |  |  |  |  |
| BIO8 | 0.569 | -0.072 | -0.116 | 0.039 | 0.604 | 0.365 | -0.018 | 1 |  |  |  |  |  |  |  |  |  |  |  |
| BIO9 | 0.945 | -0.669 | -0.066 | -0.639 | 0.471 | 0.954 | -0.709 | 0.356 | 1 |  |  |  |  |  |  |  |  |  |  |
| BIO10 | 0.815 | -0.258 | -0.475 | 0.185 | 0.980 | 0.510 | 0.056 | 0.644 | 0.613 | 1 |  |  |  |  |  |  |  |  |  |
| BIO11 | 0.932 | -0.695 | -0.012 | -0.718 | 0.398 | 0.980 | -0.781 | 0.415 | 0.979 | 0.552 | 1 |  |  |  |  |  |  |  |  |
| BIO12 | 0.711 | -0.753 | -0.233 | -0.568 | 0.282 | 0.805 | -0.669 | 0.043 | 0.815 | 0.415 | 0.780 | 1 |  |  |  |  |  |  |  |
| BIO13 | 0.715 | -0.693 | -0.093 | -0.640 | 0.215 | 0.809 | -0.713 | 0.154 | 0.815 | 0.367 | 0.805 | 0.909 | 1 |  |  |  |  |  |  |
| BIO14 | 0.691 | -0.631 | -0.296 | -0.380 | 0.409 | 0.701 | -0.484 | 0.015 | 0.762 | 0.510 | 0.690 | 0.926 | 0.755 | 1 |  |  |  |  |  |
| BIO15 | -0.528 | 0.500 | 0.488 | 0.061 | -0.517 | -0.481 | 0.190 | -0.039 | -0.512 | -0.550 | -0.445 | -0.678 | -0.364 | -0.774 | 1 |  |  |  |  |
| BIO16 | 0.728 | -0.736 | -0.139 | -0.642 | 0.234 | 0.831 | -0.724 | 0.068 | 0.841 | 0.380 | 0.818 | 0.958 | 0.979 | 0.830 | -0.474 | 1 |  |  |  |
| BIO17 | 0.701 | -0.607 | -0.325 | -0.322 | 0.467 | 0.682 | -0.430 | 0.012 | 0.757 | 0.557 | 0.674 | 0.911 | 0.731 | 0.991 | -0.800 | 0.813 | 1 |  |  |
| BIO18 | 0.553 | -0.705 | 0.025 | -0.761 | -0.043 | 0.757 | -0.813 | 0.142 | 0.686 | 0.120 | 0.731 | 0.828 | 0.927 | 0.605 | -0.230 | 0.898 | 0.561 | 1 |  |
| BIO19 | 0.716 | -0.580 | -0.285 | -0.334 | 0.468 | 0.685 | -0.432 | 0.004 | 0.784 | 0.561 | 0.689 | 0.908 | 0.744 | 0.979 | -0.769 | 0.825 | 0.986 | 0.552 | 1 |
