# Supplementary material for: Effects of climate change on the distribution of wild Akebia trifoliata
Source: Ecol Evol. 2022 Mar 23;12(3):e8714. doi: 10.1002/ece3.8714 (PMC8941373; doi:10.1002/ece3.8714)
Supplement: Supplementary file 7 — Table S2 [file ECE3-12-e8714-s009.doc]

Table S2. Climate variables and their importanc in simulating

the suitable habitat of *Akebia trifoliata*.

| Variable | Percent contribution(%) | Permutation importance(%) |
| --- | --- | --- |
| bio12 | 30.6 | 1.4 |
| bio6 | 24.3 | 3.3 |
| bio14 | 16 | 9.7 |
| bio19 | 4.5 | 3.6 |
| bio8 | 3.8 | 7.9 |
| bio4 | 3.7 | 13.5 |
| bio17 | 3.3 | 6.1 |
| bio10 | 2.7 | 4.2 |
| bio3 | 2.5 | 8.1 |
| bio11 | 1.7 | 2.5 |
| bio2 | 1.6 | 10.7 |
| bio7 | 1.2 | 0 |
| bio13 | 1.1 | 7.9 |
| bio15 | 0.7 | 5.7 |
| bio16 | 0.7 | 8.3 |
| bio9 | 0.6 | 0.6 |
| bio5 | 0.5 | 5.1 |
| bio1 | 0.2 | 0.7 |
| bio18 | 0.2 | 0.7 |
