# Supplementary material for: Effects of climate change on the distribution of wild Akebia trifoliata
Source: Ecol Evol. 2022 Mar 23;12(3):e8714. doi: 10.1002/ece3.8714 (PMC8941373; doi:10.1002/ece3.8714)
Supplement: Supplementary file 8 — Table S3 [file ECE3-12-e8714-s005.doc]

Table S3 Information of eight pairs of SSR primers

| primer | F | R | Repeating unit | *T*m（℃） |
| --- | --- | --- | --- | --- |
| P2 | CACACTTTTATAATTTGGAAAACCA | GGGAAATAGGCATCTTTGGA | (gt)_13_ | 60 |
| P5 | TCACAAATAGGGGTCTCTGTCTC | GAGGTCGTAGGTTCAAGTCCA | (tc)_5_(ac)_23_ | 58 |
| P6 | TCAATCGCTTTGGTCTTCCT | CCAAGTCAGTAGGAGGTTTGTT | (ct)_7_(ca)_9_ | 59 |
| P8 | TTTTGCGTGTGTGTGATGAA | CCACAAATATACATAAGAGGAGCAA | (gt)_6_(ga)_10_ | 57 |
| P12 | TCAACACCGTCAATGGGAGGA | AAGCAACAAAAAGAGTTGCTCATGAAT | (ca)_12_ | 53 |
| P17 | TCTGGGATTACTTGAGCAGGCA | CGCTGACGTGGTAGCGCACA | (tg)_9_(ga)_8_ | 51 |
| P21 | TCCTTCAAAAAGTGACAGGAGGA | ACTGTCAATCAACCTTGTCGCC | (gt)_16_ | 53 |
| P23 | GTGCGATCCAGACGCACCTC | ACCTGAATGGGCATCCATTTGT | (tc)_15_ | 52 |
