# Supplementary material for: Effects of climate change on the distribution of wild Akebia trifoliata
Source: Ecol Evol. 2022 Mar 23;12(3):e8714. doi: 10.1002/ece3.8714 (PMC8941373; doi:10.1002/ece3.8714)
Supplement: Supplementary file 9 — Table S4 [file ECE3-12-e8714-s001.doc]

Table S4. Suitable area of *Akebia trifoliata* in the world.

|  | | Suitable area（10^4^km^2^） | | | |
| --- | --- | --- | --- | --- | --- |
| Scenario | Year | Low | Medium | High | Total |
| current |  | 732.34 | 775.85 | 182.69 | 1690.88 |
| SSP1-2.6, | 2021 | 740.07 | 765.81 | 230.42 | 1736.30 |
|  | 2041 | 750.39 | 769.95 | 251.53 | 1771.87 |
|  | 2061 | 728.93 | 824.65 | 261.18 | 1814.76 |
|  | 2081 | 804.38 | 742.53 | 255.94 | 1802.85 |
| SSP2-4.5 | 2021 | 799.57 | 753.76 | 252.24 | 1805.57 |
|  | 2041 | 776.21 | 765.65 | 272.26 | 1814.12 |
|  | 2061 | 740.21 | 728.23 | 299.41 | 1767.85 |
|  | 2081 | 788.47 | 696.39 | 325.95 | 1810.81 |
| SSP3-7.0 | 2021 | 718.19 | 746.79 | 225.11 | 1690.09 |
|  | 2041 | 784.22 | 712.14 | 257.97 | 1754.33 |
|  | 2061 | 872.49 | 693.86 | 311.71 | 1878.06 |
|  | 2081 | 1080.50 | 670.42 | 321.77 | 2072.69 |
| SSP5-8.5 | 2021 | 762.47 | 728.23 | 247.00 | 1737.70 |
|  | 2041 | 807.87 | 768.96 | 288.61 | 1865.44 |
|  | 2061 | 1136.01 | 699.67 | 363.80 | 2199.48 |
|  | 2081 | 1094.71 | 663.52 | 349.18 | 2107.41 |
