# Supplementary material for: Effects of climate change on the distribution of wild Akebia trifoliata
Source: Ecol Evol. 2022 Mar 23;12(3):e8714. doi: 10.1002/ece3.8714 (PMC8941373; doi:10.1002/ece3.8714)
Supplement: Supplementary file 10 — Table S5 [file ECE3-12-e8714-s002.doc]

Table S5. Suitable area of *Akebia trifoliata* in China.

|  | | Suitable area（10^4^km^2^） | | | |
| --- | --- | --- | --- | --- | --- |
| Scenario | Year | Low | Medium | High | Total |
| current |  | 80.61 | 102.24 | 9.16 | 192.01 |
| SSP1-2.6 | 2021 | 80.61 | 102.23 | 9.16 | 192.00 |
|  | 2041 | 60.12 | 102.92 | 25.87 | 188.91 |
|  | 2061 | 45.46 | 104.51 | 39.72 | 189.69 |
|  | 2081 | 45.35 | 104.11 | 41.34 | 190.80 |
| SSP2-4.5 | 2021 | 42.39 | 108.76 | 49.86 | 201.01 |
|  | 2041 | 58.82 | 108.35 | 21.49 | 188.66 |
|  | 2061 | 39.51 | 127.80 | 41.82 | 209.13 |
|  | 2081 | 35.41 | 106.48 | 66.52 | 208.41 |
| SSP3-7.0 | 2021 | 40.63 | 88.77 | 93.10 | 222.50 |
|  | 2041 | 59.71 | 100.85 | 21.20 | 181.76 |
|  | 2061 | 47.69 | 107.03 | 41.99 | 196.71 |
|  | 2081 | 64.58 | 86.93 | 81.76 | 233.27 |
| SSP5-8.5 | 2021 | 74.79 | 82.04 | 88.10 | 244.93 |
|  | 2041 | 41.33 | 124.56 | 32.10 | 197.99 |
|  | 2061 | 33.77 | 110.67 | 54.14 | 198.58 |
|  | 2081 | 73.53 | 84.77 | 95.51 | 253.81 |
