# Supplementary material for: Effects of climate change on the distribution of wild Akebia trifoliata
Source: Ecol Evol. 2022 Mar 23;12(3):e8714. doi: 10.1002/ece3.8714 (PMC8941373; doi:10.1002/ece3.8714)
Supplement: Supplementary file 11 — Table S6 [file ECE3-12-e8714-s011.doc]

Table S6. Suitable area of *Akebia trifoliata* in the past.

| Suitable area（10^4^km^2^） | | | | |
| --- | --- | --- | --- | --- |
| Scenario | Low | Medium | High | Total |
| The Last Interglacial | 430.38 | 206.62 | 24.15 | 661.15 |
| The Last Glacial Maximum | 598.58 | 243.04 | 69.20 | 910.81 |
| The Mid Holocene | 563.91 | 588.23 | 443.88 | 1596.03 |
